# Advanced Signal Processing Methods for Partial Discharge Analysis: A Review

**DOI:** 10.3390/s25237318

**Published:** 2025-12-01

**Authors:** He Wen, Mohamad Sofian Abu Talip, Mohamadariff Othman, S. M. Kayser Azam, Mahazani Mohamad, Mohd Faisal Ibrahim, Hamzah Arof, Ahmad Ababneh

**Affiliations:** 1 Department of Electrical Engineering, Faculty of Engineering, Universiti Malaya, Kuala Lumpur 50603, Malaysia; 23104961@siswa.um.edu.my (H.W.); mohamadariff@um.edu.my (M.O.); kayser@um.edu.my (S.M.K.A.); ahamzah@um.edu.my (H.A.); 2 Department of Electrical, Electronic and Systems Engineering, Faculty of Engineering and Built Environment, Universiti Kebangsaan Malaysia, Bangi 43600, Malaysia; faisal.ibrahim@ukm.edu.my; 3 College of Engineering and Technology, American University of the Middle East, Egaila 54200, Kuwait; ahmad.ababnah@aum.edu.kw

**Keywords:** partial discharge, signal processing, time-frequency analysis, artificial intelligence, fault diagnosis

## Abstract

This paper comprehensively reviews advanced signal processing methods for partial discharge (PD) analysis, covering traditional time-frequency techniques, wavelet transform, Hilbert–Huang transform, and artificial intelligence-based methods. This paper critically examines the principles, advantages, limitations, and applicable scenarios of each method. A key contribution of this review is the systematic comparison of these methods, highlighting their evolution and complementary roles in processing non-stationary and noisy PD signals. However, a significant gap in current research remains the lack of standardized, explainable, and embeddable AI solutions for real-time, fine-grained PD classification. Future trends point to hybrid approaches and edge AI systems that combine physical insights with lightweight deep learning models to improve diagnostic accuracy and deployability.

## 1. Introduction

In modern power systems, with the widespread application of high-voltage equipment, the research and monitoring of partial discharge (PD) phenomena have become increasingly important. Partial discharge refers to arc discharge caused by local increase in electric field strength in a local area of an insulating material or electrical equipment. It usually occurs in tiny areas such as insulation defects, bubbles or interfaces. Although partial discharge does not immediately lead to complete insulation breakdown, the negative impact it brings is continuous and gradually accumulated [1].

**Figure 1:**
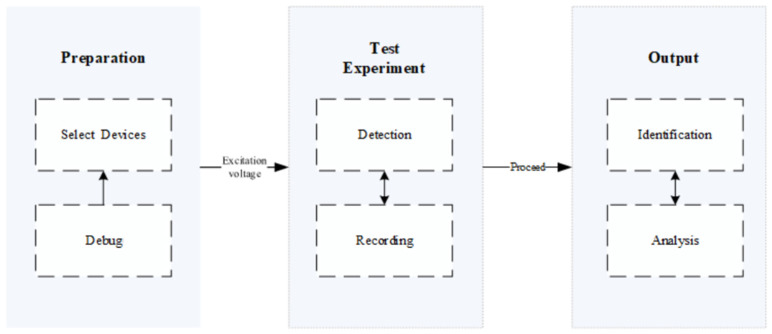
Standard Procedure for Partial Discharge Detection Experiment.

In severe cases, it may lead to equipment failure, operational safety hazards and economic losses. The mechanism of partial discharge is inseparable from the effect of high-voltage electric fields. When the electric field strength exceeds the local breakdown strength of the insulating material, electrons are accelerated in the defect area and form tiny plasmas, this process is accompanied by continuous energy release, resulting in the gradual degradation of the physical and chemical properties of the insulating material [2,3]. This degradation not only affects the insulation performance, but may also accelerate the aging process of the equipment and cause more serious failures, such as equipment failure or system power outages [4]. Therefore, early detection of partial discharge phenomena is crucial. We can understand the general process of partial discharge detection based on Figure 1.

- First, select appropriate partial discharge detection equipment based on the type and requirements of the tested equipment, and ensure that the tested equipment is in normal working condition during the testing process.
- Then apply excitation voltage, gradually increase the voltage to the rated level, observe whether there is partial discharge phenomenon, that is, monitor the partial discharge signal in real time through detection equipment, usually including amplitude, phase, and frequency, and then record the data.
- Then perform some specific processing on the signal data, such as filtering, denoising, etc. Then analyze and determine the discharge type to obtain the health status of the equipment insulation.
- Finally, generate a test report for technical personnel to use.

Timely monitoring and analysis of the characteristics and behaviors of partial discharge can provide an important basis for equipment maintenance and troubleshooting, thereby reducing the operational risks caused by equipment failures [5].

At the same time, the development of partial discharge monitoring technology makes real-time monitoring possible, which can identify potential problems at an early stage and take corresponding maintenance measures to effectively extend the service life of equipment and improve the overall reliability of the power system [6]. As partial discharge monitoring technology gradually matures, the importance of analyzing PD signals has become increasingly prominent.

**Figure 2:**
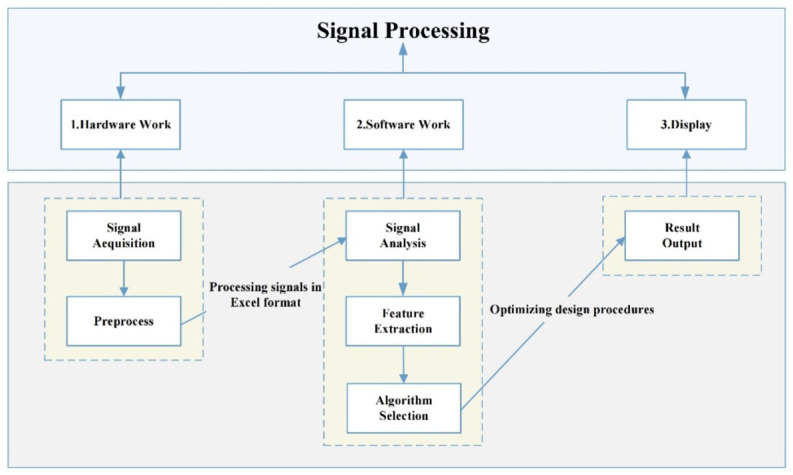
Workflow of Partial Discharge Signal Processing System.

As shown in Figure 2, the processing flow of partial discharge signals can include obtaining a certain discharge signal source through sensors, and then performing signal preprocessing such as denoising, filtering, and amplification to improve signal quality and lay a good foundation for subsequent analysis. After preparing for these tasks, signal analysis can be carried out by calculating some characteristic parameters such as peak value, mean value, etc. to preliminarily determine the signal characteristics.

Then, the algorithm is used to further extract its key features such as frequency spectrum, phase features, etc., and select the most representative features for subsequent recognition. After obtaining these signal characteristics, we select AI algorithms based on their features to ensure accuracy, and finally output the results in the form of charts for intuitive understanding.

A thorough understanding of the characteristics and changing laws of partial discharge signals enables us to better judge the health status of electrical equipment and formulate scientific maintenance strategies. Therefore, in-depth research on partial discharge signals not only has important theoretical significance, but also provides strong support for application practice, laying the foundation for the safe and efficient operation of power systems.

The development of partial discharge signal processing technology has followed a clear technological trajectory. In the 1990s, time-domain and Fourier-based methods dominated, providing basic but limited analytical capabilities [7,8,9]. In the early 2000s, wavelet transform emerged in PD analysis due to its excellent time-frequency localization capabilities and became an effective tool for processing non-stationary signals [10,11]. By the 2010s, fully adaptive methods such as empirical mode decomposition (EMD) and Hilbert–Huang transform (HHT) became increasingly prominent due to their ability to handle nonlinear and transient signals [12]. In recent years, the emergence of artificial intelligence and deep learning (e.g., Convolutional Neural Network (CNN), Recurrent Neural Network (RNN)) has completely changed the PD analysis paradigm, enabling end-to-end feature learning and automatic diagnosis, and achieving breakthrough progress in classification accuracy [13]. However, in practical engineering applications, the monitoring and analysis of PD signals face numerous challenges. First, the complex electromagnetic environment at the site makes PD signals easily obscured by strong background noise (such as carrier communication and random pulse interference), posing a primary challenge to reliable signal extraction. Second, PD signals themselves are non-stationary and transient, and different types of discharges (such as internal discharge and surface discharge) have varying physical mechanisms and time-frequency characteristics, making it difficult for general signal processing models to achieve accurate identification. Furthermore, power equipment condition monitoring demands high real-time performance, but there is a significant contradiction between limited on-site computing resources (especially on embedded edge devices) and complex signal processing algorithms. These challenges in engineering practice are the fundamental driving force behind the evolution of signal processing methods from traditional analysis to adaptive time-frequency processing, and then to an intelligent hybrid paradigm. This review traces this evolution and systematically compares each paradigm to identify its advantages, limitations, and future synergies.

### Research Contributions

We aim to make the following contributions by reviewing existing literature on partial discharge signal processing techniques:

1. A Unified Comparison Framework: Moving beyond sequential descriptions, we propose a structured, multi-dimensional comparison framework for PD signal processing methods. This framework categorizes methods based on core engineering objectives, providing practical guidance for method selection in specific scenarios.
2. An evolutionary path analysis: We critically synthesize the development of these methods, which is not a simple timeline, but an evolutionary process driven by the practical limitations of previous methods. This explains why the field has evolved from Fourier to wavelets, adaptive decomposition, and ultimately to AI.
3. A forward-looking perspective on embedded intelligence: We identify and analyze the key gap between the high precision of AI and the need for real-time embedded deployments. Our contribution lies in proposing a specific gap analysis and a practical selection framework (Section Unified PD Signal Processing Technology Selection Framework) that prioritizes the development of physically embedded, lightweight, and interpretable AI solutions for edge devices—an area not fully covered by previous reviews.

## 2. Partial Discharge Types

Analyzing the types and characteristics of partial discharge (PD) signals is a critical step in electrical insulation testing and diagnosis. Different types of PD signals, such as internal, surface, and air-gap discharge, have distinct characteristics, reflecting the different physical mechanisms of insulation defects. Table 1 shows the processing methods for different partial discharge signals. Common signal types include the following:

- Corona Partial Discharge: It occurs when the electric field strength exceeds the dielectric strength of air, resulting in ionization around the conductor.
- Internal Partial Discharge: It usually occurs inside electrical equipment, especially inside the insulation of electrical equipment, such as transformers, switchgear, insulation materials, etc. Internal discharge may cause partial discharge due to aging, cracks or defects in insulation materials, which may cause equipment failure.
- Surface Partial Discharge: It occurs when discharge occurs on the surface of an insulator or between a conductor and an insulator. Surface discharge often occurs when the insulation surface is stained, damp, or aged. It can degrade the insulation performance of electrical equipment and, in severe cases, lead to equipment failure.
- Gap discharge: Gap discharge occurs when the electric field strength in a gas, vacuum, or other insulating medium is high enough to cause the dielectric (such as air) to break down, leading to discharge. This phenomenon typically occurs in air gaps or gaps between insulators within electrical equipment. Gap discharge requires a certain voltage (i.e., the breakdown voltage), and once it occurs, it can damage the equipment.

## 3. Review Methodology

To ensure a comprehensive and objective review of the literature on partial discharge signal processing, we employ a systematic literature search and screening process. The main objective of this review is to identify and analyze relevant scientific literature reporting advanced signal processing techniques for PD analysis, with particular focus on the technological evolution path from traditional methods to artificial intelligence and hybrid paradigms.

### 3.1. Database and Retrieval Strategies

Literature searches were primarily conducted in core scientific databases such as IEEE Xplore, ScienceDirect (Elsevier), Web of Science, and Scopus. The search strategy employed a combination of keywords and Boolean logic operators to capture relevant research. Core keywords included: “partial discharge,” “PD signal processing,” “time-frequency analysis,” “wavelet transform,” “empirical mode decomposition,” “Hilbert-Huang transform,” “artificial intelligence,” “deep learning,” “CNN,” “RNN,” and “edge computing,” combined with terms such as “condition monitoring,” “fault diagnosis,” “denoising,” and “pattern recognition.”

### 3.2. Literature Screening Criteria

The preliminary search results were filtered according to the following criteria:

1. Publication Time: The search focuses on literature published between 1990 and 2024, with a particular emphasis on foundational research from the 1990s to the 2000s, and significant advancements since 2010, especially the AI-based research boom since 2022.
2. Document Type: Peer-reviewed journal articles, conference papers, and high-quality review articles are prioritized. Books, patents, and non-peer-reviewed technical reports are excluded.
3. Technical Focus: Literature primarily researching novel or critically applicable PD signal processing algorithms is included. Articles focusing solely on sensor design, without signal processing, or purely commercial application reports lacking methodological insights are excluded.
4. Application Background: To provide a balanced perspective, this review covers research based on laboratory-simulated PD (using common defect models such as pin-plate, air gap, and surface discharge) and field measurements (from critical power equipment such as transformers, gas-insulated switchgear (GIS), and power cables). At the same time, signals obtained through mainstream sensing technologies such as high-frequency current transformers (HFCTs), ultra-high frequency (UHF) sensors, and acoustic emission sensors were also considered.

### 3.3. Scope and Balance Description

Through the above process, this review aims to cover diverse technologies, equipment, and application scenarios. At the technical level, we have included a balanced approach to signal processing research based on different sensing technologies such as HFCT, UHF, and acoustics. In terms of applications, the review covers both studies validating algorithm performance in controlled laboratory environments and applications facing challenges in real-world industrial settings. The types of equipment covered are comprehensive, with a focus on transformers, GIS, and power cables—the most critical components in PD monitoring. This multi-dimensional coverage strategy ensures that this review provides a panoramic and balanced comprehensive assessment of PD signal processing technologies.

## 4. Methods

A thorough understanding of the characteristics and changing laws of partial discharge signals enables us to better judge the health status of electrical equipment and formulate scientific maintenance strategies. Therefore, in-depth research on partial discharge signals processing not only has important theoretical significance, but also provides strong support for application practice, laying the foundation for the safe and efficient operation of power systems. Next this paper will introduce the specific applicability, advantages and disadvantages of different methods according to the complexity of the signal type.

### 4.1. Time Domain Analysis

Time-domain analysis identifies and characterizes partial discharge (PD) activity by directly examining the timeline characteristics of the signal, including its waveform, amplitude, statistical distribution, and pulse intervals. This method, due to its intuitiveness and high computational efficiency, has been widely used in preliminary diagnosis, pulse identification, and trend analysis. The effectiveness of time-domain analysis depends largely on extracting highly discriminative feature parameters from the raw signal. These parameters are the core vehicle for quantifying the morphology, statistical characteristics, and evolution of PD pulses, forming a bridge between the raw signal and advanced diagnostic decisions. Time-domain analysis methods identify and characterize discharge activity by directly analyzing the waveform, amplitude distribution, intervals between adjacent pulses, and phase characteristics of the PD signal. These analyses help determine the type, intermittency, periodicity, and relationship between the discharge and the applied voltage [20]. Furthermore, time-domain analysis employs envelope detection and statistical feature extraction, such as mean, variance, skewness, and kurtosis, to identify regular trends in the signal, providing a basis for pattern recognition [20].

#### 4.1.1. Core Time Domain Characteristic Parameters

Table 2 lists the time domain characteristic parameters commonly used in the field of partial discharge.

**Figure 3:**
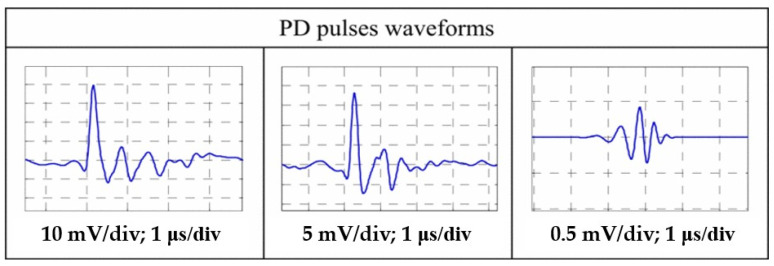
Partial discharge pulse waveform (measured using a high-frequency current sensor HFCT) [21]. The figure shows the partial discharge pulse waveforms observed at different voltage sensitivities (10 mV/division, 5 mV/division, and 0.5 mV/division) at a time base of 1 μs/second. Measurement conditions included a sampling rate of 100 MS/s, a bandwidth of 0.5–30 MHz, and pulse signals acquired via a 50 Ω matched terminal.

Figure 3 shows a typical partial discharge (PD) pulse current waveform captured using a broadband measurement system. The figure consists of three waveforms acquired at the same time scale (1 μs/div) and different voltage sensitivities (from left to right: 10 mV/div, 5 mV/div, and 0.5 mV/div).

These three waveforms together reveal the key characteristics of an actual PD pulse: an extremely steep rising edge, a sharp peak, and a high-frequency decaying oscillation following the peak caused by resonance in the measurement circuit. Different sensitivity settings provide multiple perspectives: the left image shows the pulse’s global profile and the entire decaying oscillation; the right image clearly demonstrates the pulse’s rising edge in high resolution, making it suitable for accurately measuring key parameters such as rise time; and the center image provides an intermediate-scale perspective.

This set of waveforms confirms that PD pulses in actual measurements typically appear as high-frequency decaying oscillations with microsecond pulse widths, a very classic and representative waveform morphology under broadband measurement conditions.

#### 4.1.2. Method Corresponding to the Parameter

Different parameters serve different analytical purposes and correspond to specific processing methods. Table 3 systematically summarizes typical methods and their applications based on these core parameters.

Furthermore, using RBA (Ramping Behavior Analysis) to extract and classify the acquired time-domain parameters can effectively identify PD pulses, extract PD event features, and analyze PD event trends and patterns [28]. Pulse waveform identification can effectively distinguish between different discharge sources. For example, by examining features such as pulse repetition rate and pulse amplitude, corona discharge and internal discharge can be effectively distinguished, especially when both occur simultaneously [29]. Furthermore, through correlation analysis between high-frequency and ultra-high-frequency signals, time-domain analysis can significantly improve the signal-to-noise ratio by 15–20 dB, thereby helping to distinguish PD signals from interfering noise [30]. Finally, short-term feature analysis techniques, such as short-time energy and zero-crossing counts, have been shown to effectively distinguish partial discharge signals from noise in both laboratory and field environments [31].

In summary, time-domain analysis plays a key role in PD signal processing, providing a direct and intuitive approach to extracting important time-domain features. However, its limitations are also significant, primarily including sensitivity to noise and limited information extraction [32]. In strong noise environments or when complex discharge patterns need to be analyzed, it is often necessary to combine it with frequency-domain or time-frequency analysis methods.

### 4.2. Fourier Transform (FT)

Fourier transforms and their evolutionary methods provide core tools for analyzing the frequency content of partial discharge signals. This section provides a conceptual review of these methods, focusing on their performance, applicable scenarios, and limitations in PD analysis.

#### 4.2.1. Method Overview and Evolution

The Fourier transform (FT) is a mathematical tool that converts signals from the time domain to the frequency domain. Its core is to decompose complex signals into a combination of sinusoidal waves of different frequencies, thereby revealing their global spectral characteristics. For discrete signals, the fast Fourier transform (FFT) has become the basis of spectrum analysis due to its excellent computational efficiency. In PD analysis, FFT can effectively identify stable resonant frequency components in the signal and design digital filters based on this to suppress interference at specific frequencies [33,34,35]. However, the standard FFT cannot provide information about the time-varying frequency components. This limitation is particularly prominent when dealing with non-stationary PD transient pulses. The short-time Fourier transform (STFT) provides the time-frequency distribution of the signal by introducing a sliding time window, thereby capturing the transient characteristics of the PD pulse [18,36]. As an application of STFT, the quadratic short-time Fourier transform is used to extract the time-frequency distribution of the PD pulse energy to understand the dynamic change process of the discharge event [37]. As an extension of the STFT, the local polynomial Fourier transform (LPFT) improves the time-frequency aggregation through polynomial modeling, and is particularly good at revealing the fine features of the low and medium frequency parts of the PD signal [38,39].

As a generalized form of the traditional Fourier transform, the fractional Fourier transform (FRFT) transforms the signal into the fractional order domain between time and frequency. For certain specific forms of PD signals (such as pulses with linear frequency modulation characteristics), FRFT can provide stronger energy aggregation and analysis capabilities than traditional methods [40,41].

In the process of seeking higher-resolution time-frequency analysis, other methods have also emerged. For example, the S transform, as a time-frequency analysis method that can provide adaptive resolution, has been applied to the analysis of power cable PD signals and has been compared with traditional methods such as STFT [42,43]. In addition, the idea of combining wavelet packet transform with generalized morphological filters also reflects the exploration of integrating frequency domain analysis (wavelet packets) with advanced filtering technology to improve PD monitoring effects [44].

#### 4.2.2. Performance Comparison and Application Scenarios

Different Fourier transform methods exhibit widely varying performance when processing PD signals, and the choice of which method to use is highly dependent on the specific noise environment, signal characteristics, and analysis objectives. Table 4 provides a comparison of the core characteristics and performance of these methods.

Summary and Selection Guide: The FFT is the preferred tool for analyzing the global spectrum of a signal and filtering fixed-frequency noise, but its value in PD analysis is typically fundamental and supplementary. The STFT introduces temporal localization capabilities based on the FFT, providing a practical compromise for preliminary time-frequency analysis of PD transients. However, its fixed resolution is an inherent bottleneck. The LPFT and FRFT represent more advanced efforts to overcome the limitations of the STFT. The LPFT increases resolution to capture finer features, while the FRFT changes the transform domain to better match the inherent structure of certain complex PD signals.

In practical applications, these methods are often used as front-ends for feature extraction, combined with classifiers such as artificial intelligence, rather than as standalone diagnostic tools. The choice requires a balance between computational efficiency, required time-frequency resolution, and signal characteristics.

### 4.3. Wavelet Transform (WT)

Compared to FT, Wavelets transform the signal by replacing the infinitely long trigonometric bases of the Fourier transform with localized, rapidly decaying wavelet bases. unlike Fourier transform, which only has frequency ω, wavelet transform has two variables: scale α and translation τ. Scale α controls the scaling of the wavelet function, while translation τ controls the translation of the wavelet function. What is different from the Fourier transform is that this not only tells us that the signal has such a frequency component, but also knows its specific location in the time domain. When we translate and multiply the signal at each scale, we know which frequency components the signal contains at each location. In other words, wavelet transform solves the problem of unstable signals.

Fourier transform can only obtain a frequency spectrum, while wavelet transform can obtain a time-frequency spectrum. Wavelet transform is a powerful signal processing technology that analyzes signals in both the time domain and the frequency domain by decomposing them into linear combinations of wavelet functions at different scales. It is particularly effective for processing non-stationary signals, showing significant advantages in partial discharge (PD) signal analysis.

Wavelet transform decomposes the signal into approximate components and detail components of different scales through multi-resolution analysis, thereby effectively capturing the local characteristics of the signal. Compared with traditional time domain analysis or Fourier transform, its multi-scale characteristics enable the signal to be observed at different resolutions and can simultaneously identify different frequency components and transient details in the signal. This characteristic is crucial for analyzing partial discharge (PD) signals containing multiple frequency components and complex waveforms [49,50]. In PD signal processing, the advantages of wavelet transform are mainly reflected in three aspects:

- Excellent noise removal capabilities: Wavelet transform can selectively retain signal components and suppress noise through wavelet filter design and threshold processing based on Pareto optimization. Its performance is superior to traditional time-domain methods or filtering techniques, especially under low signal-to-noise ratio conditions [51,52,53,54]. By optimizing wavelet families such as Daubechies, Symlets, and Coiflets, the signal-to-noise ratio (SNR) can be significantly improved, thereby enhancing the detection performance of PD signals in high-noise environments [52,54,55].
- Excellent time-frequency localization capability: The wavelet transform can provide both time-domain and frequency-domain information of a signal, making it ideal for analyzing non-stationary transient signals such as PD, as well as signals whose statistical characteristics vary over time. This dual-domain analysis capability gives it a strong advantage in transient event detection, overcoming the limitation of the traditional Fourier transform, which loses time-domain details [50,56].
- Flexibility and adaptability: The flexibility of the wavelet transform lies in the ability to select the most appropriate wavelet basis function based on the characteristics of a specific PD signal, thereby achieving more targeted analysis and improving detection accuracy. Unlike traditional methods, it even allows for custom wavelet bases to better match signal characteristics [52,55].

However, the performance of the wavelet transform depends heavily on parameters such as the choice of wavelet basis, the number of decomposition levels, and the threshold function [57]. To fully tap its potential, deep expertise and comprehensive judgment are required. Under extremely low signal-to-noise ratio conditions, a single wavelet transform may not be sufficient to meet the requirements. Therefore, researchers often combine it with techniques such as empirical mode decomposition (EMD). This hybrid approach has been shown to effectively improve noise suppression while better preserving the signal’s information integrity in low signal-to-noise ratio environments [58]. In summary, despite the challenges of parameter selection, wavelet transform provides a comprehensive and detailed analysis method for partial discharge signals due to its significant advantages in denoising, time-frequency analysis and flexibility, making it an alternative to traditional signal processing techniques.

#### Wavelet Packet Transform (WPT)

The discrete wavelet transform (DWT) recursively decomposes low-frequency components (approximation coefficients) while disregarding high-frequency components (detail coefficients). The WPT extends this approach, providing a more refined and versatile analysis scheme capable of equally in-depth recursive decomposition of high-frequency detail components. This ability to perform multi-resolution analysis across the entire frequency band gives it an advantage over the standard DWT in extracting fine features from partial discharge (PD) signals amidst complex noise backgrounds.

- Enhanced signal decomposition and noise reduction capabilities: The more detailed signal decomposition provided by WPT enables better noise separation and signal clarity. This is particularly beneficial for distinguishing PD pulses with similar frequency characteristics to noise [59,60]. Research has shown that improved WPT methods can effectively extract PD signals from complex noisy environments, such as power cables, and exhibit excellent noise reduction performance [59].
- Synergistic integration with other technologies: Combining it with principal component analysis (PCA) can effectively suppress noise while preserving key PD features [61]. Combined with singular value decomposition (SVD), it can specifically eliminate periodic narrowband interference [60]. A combined Kalman filter and WPT denoising method has been shown to significantly improve the signal-to-noise ratio while reducing waveform distortion [62].

Furthermore, the choice of mother wavelet is crucial to the effectiveness of WPT. Selecting the optimal wavelet basis based on metrics such as the cross-correlation coefficient can significantly improve noise suppression and signal detection [52,63]. Previous studies have experimentally compared the effectiveness of different wavelet families, such as Symlet and Coiflet, in PD denoising. The results show that the selection depends on the specific noise characteristics, with varying success rates [64].

### 4.4. Empirical Mode Decomposition (EMD) and Hilbert–Huang Transform (HHT)

The Hilbert–Huang transform (HHT) is an adaptive time-frequency analysis method specifically designed for analyzing nonlinear and nonstationary signals [65,66]. It is essentially a two-stage process: first, the signal is adaptively decomposed into a set of intrinsic mode functions (IMFs) using empirical mode decomposition (EMD); second, each IMF is Hilbert transformed to obtain a high-resolution time-frequency energy distribution (called the Hilbert spectrum) [67,68]. Because it does not rely on prior basis functions, HHT exhibits unique advantages in processing complex transient signals such as PD.

#### 4.4.1. Empirical Mode Decomposition (EMD) Process

**Figure 4:**
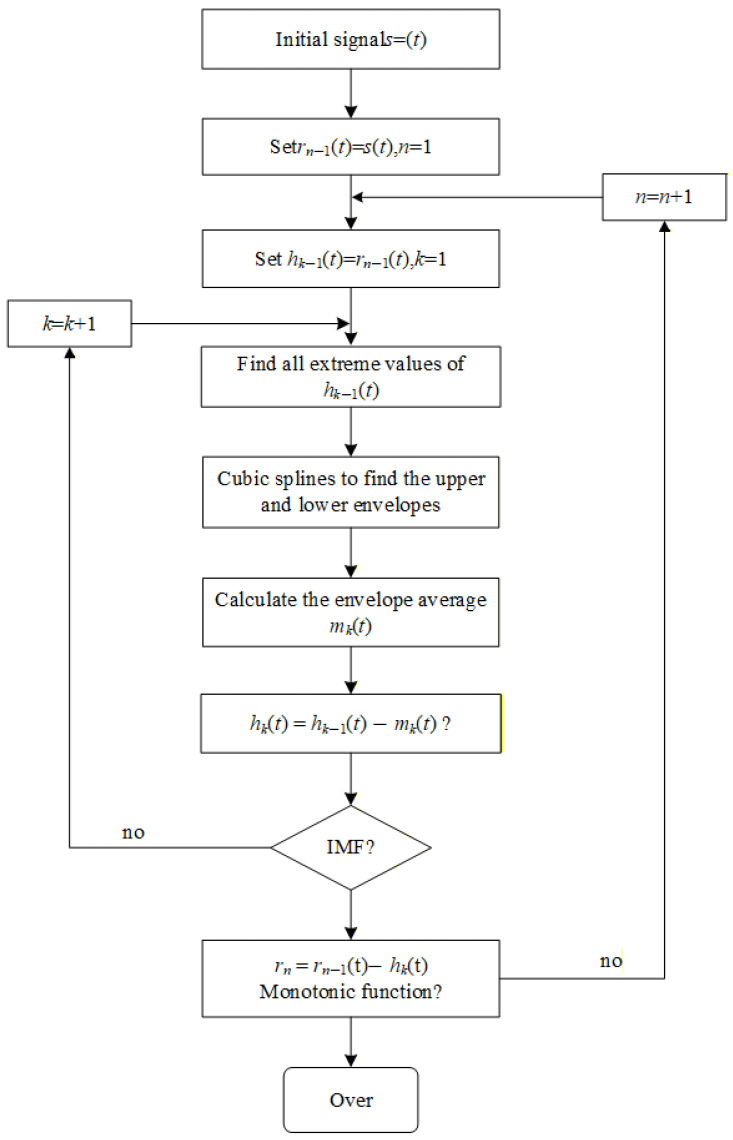
Iterative process of Empirical Mode Decomposition (EMD) algorithm for processing partial discharge signals.

EMD is the basis of HHT, which aims to decompose the original signal into a series of intrinsic mode functions (IMFs) through a process called “sieving”. The specific process is shown in Figure 4. Each IMF must meet two conditions: (1) the number of extreme points is equal to or differs from the number of zero crossings by at most one; (2) the mean of the envelope defined by the local maxima and local minima is zero [53]. The sieving process first identifies all the extreme points of the signal and fits the upper and lower envelopes, respectively, using cubic spline interpolation. The difference between the original signal and the mean of the upper and lower envelopes is called the intermediate signal. This process is repeated until the intermediate signal meets the IMF conditions, thus obtaining the first IMF. This IMF is separated from the original signal to obtain the residual, and the above process is repeated on the residual until the residual is a monotonic function or a constant. Finally, the original signal is represented as the sum of several IMFs and a residual [69].

EMD and its improved algorithms (such as EEMD) can effectively reduce signal noise by screening and retaining IMFs that reflect the main signal characteristics and eliminating noise-dominated components [70,71]. In the presence of strong noise, combining energy threshold and sensitivity function analysis can be used to identify key IMFs and ensure accurate signal reconstruction [70,72].

#### 4.4.2. From EMD to Hilbert Spectrum

After obtaining the IMFs through EMD, the second stage of HHT is to perform a Hilbert transform on each IMF to calculate its instantaneous frequency and instantaneous amplitude. Subsequently, the instantaneous frequencies and amplitudes of all IMFs are combined on the time-frequency plane to obtain the Hilbert spectrum, which clearly reveals the subtle evolution of the signal energy in time and frequency [66,67].

Compared to the wavelet transform, which requires pre-set basis functions, the fully adaptable nature of HHT makes it superior in extracting detailed features of PD signals. Research has shown that features extracted using HHT (such as the time-frequency entropy vector) often outperform traditional wavelet transforms in clustering and classification performance in PD pattern recognition and fault diagnosis [66,67,72]. The recognition and classification accuracy of PD features can be further improved by machine learning techniques [65,73] or combining HHT with fractal parameter analysis [74,75].

#### 4.4.3. Improved Algorithms and Developments of EMD

Although the basic EMD algorithm is powerful, problems such as modal aliasing and sensitivity to noise have led to the emergence of a series of improved algorithms. Table 5 systematically compares the characteristics of several mainstream algorithms in the EMD family.

The table shows that the transition from EMD to EEMD and then to CEEMDAN involves a continuous trade-off and optimization process between computational efficiency, decomposition accuracy, and noise immunity. The choice should be made based on the specific PD signal characteristics, the noise environment, and the accuracy and speed requirements of the task. These improved algorithms (such as CEEMDAN and variational mode decomposition (VMD)) effectively overcome the shortcomings of the original EMD through different mechanisms and, combined with techniques such as wavelet threshold denoising, further improve the performance of processing PD signals in low SNR environments [58,76,77,78,79]. Application cases have shown that EMD-based methods can significantly improve the SNR and clarity of PD signals in practical scenarios such as power equipment switchgear and gas-insulated transmission lines [72,80].

### 4.5. Comprehensive Comparison and Selection Guide for Time-Frequency Analysis Methods

**Figure 5:**
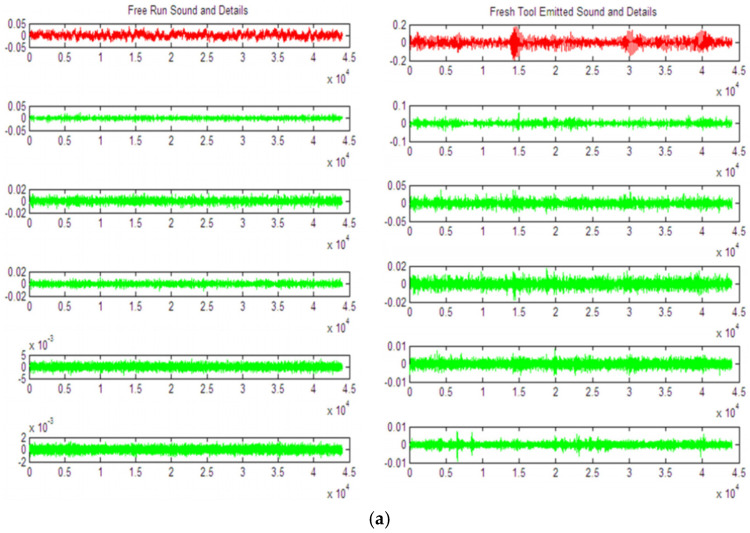

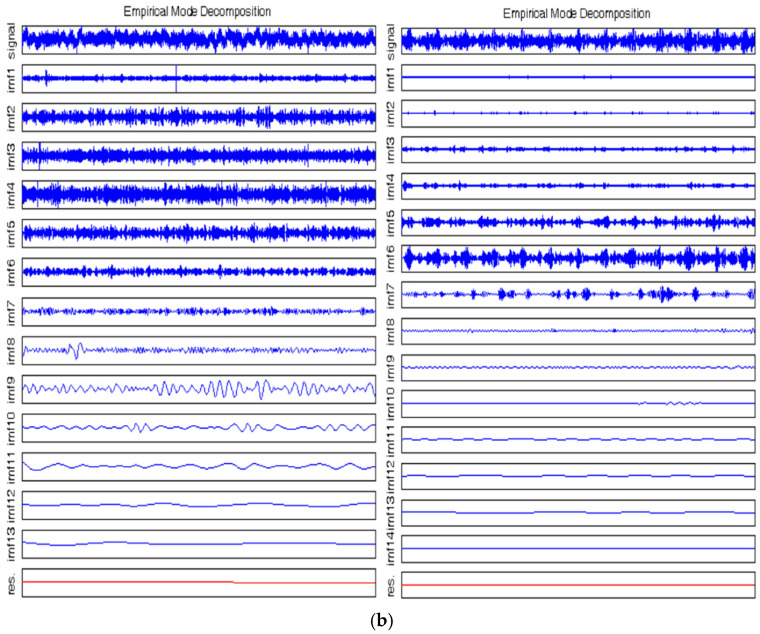
Comparison of Time-Frequency Analysis Based on Discrete Wavelet Transform (DWT) and Empirical Mode Decomposition (EMD) [81]. (**a**) Discrete wavelet Transform (DWT) analysis of a tool sound signal, showing its regular but fixed-scale decomposition coefficients. (**b**) Empirical mode decomposition (EMD) analysis of the same signal, showing its adaptive generation of a variable number of intrinsic mode functions (IMFs). This comparison intuitively illustrates the core difference between the DWT’s a priori, fixed structure, and the EMD’s a posteriori, adaptive structure.

**Figure 6:**
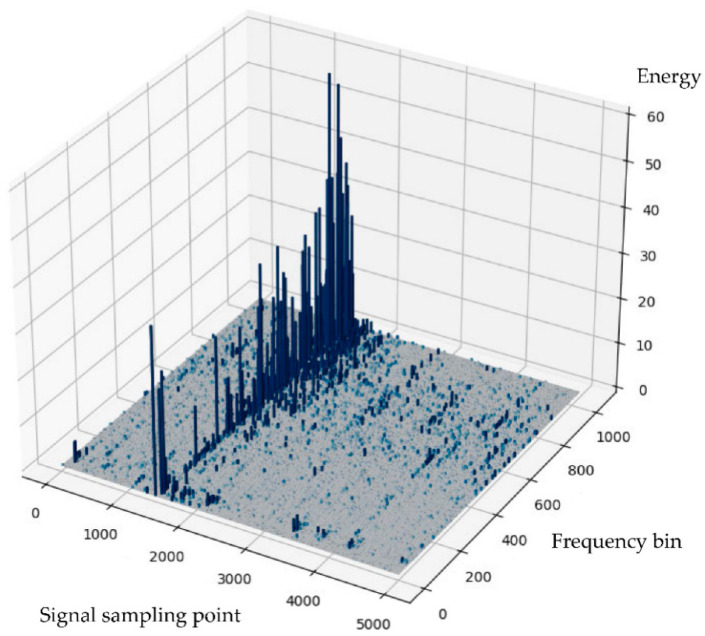
Time-frequency energy three-dimensional distribution of partial discharge signal [82]. The Hilbert spectrum is obtained by HHT, which can clearly reveal the evolution of signal energy with time and frequency. It does not have the energy diffusion problem caused by fixed basis functions in traditional methods, thus providing more accurate time-frequency positioning.

The previous article systematically explained the application of wavelet transform (WT), empirical mode decomposition (EMD), and Hilbert–Huang transform (HHT) to partial discharge (PD) signal analysis. These methods represent different technical philosophies for processing nonstationary, nonlinear PD signals: the wavelet transform provides a structured time-frequency analysis framework using predefined basis functions, empirical mode decomposition uses a fully data-driven adaptive approach to decompose the signal, and the Hilbert–Huang transform further provides high-resolution time-frequency energy representation. Each method exhibits unique advantages and limitations in terms of feature extraction capability, computational efficiency, noise robustness, and applicability to specific PD signal morphologies. To support the rational selection and effective application of these methods in engineering practice and scientific research, this section will systematically compare the core characteristics of these signal processing methods based on the aforementioned analysis and propose guiding principles for method selection based on practical application scenarios, providing a theoretical basis for building efficient and reliable PD diagnostic systems. To intuitively demonstrate the output differences between different methods when processing actual signals, we cite the classic case of tool wear acoustic emission signal analysis by Joseph et al. [81] and Wang et al. [82] for illustration. Figure 5 and Figure 6 provide a visual comparison.

Table 6 systematically compares the core characteristics of the three from principles to applications.

Summary and Selection Guide: The choice of method depends on the specific diagnostic objectives, signal characteristics, and system resources: When processing speed, stability, and strong noise suppression are prioritized (such as in online monitoring systems), DWT is a more reliable and efficient choice. Its regular structure and mature algorithms provide a solid foundation for real-time processing. For highly complex, nonlinear PD signals, and when signal adaptability is extremely high, EMD can be considered. However, attention should be paid to its stability issues, and it is generally recommended to combine it with other denoising algorithms to form a hybrid strategy. When the ultimate goal of analysis is high-precision fault classification and diagnosis, and sufficient computing resources are available to obtain the most detailed time-frequency features, HHT can provide the most discriminative feature input. It is suitable for offline, in-depth diagnostic analysis scenarios.

In summary, the transition from WT to EMD/HHT reflects a fundamental trade-off between computational efficiency and robustness, and between signal adaptation and feature resolution accuracy. There is no single best method, only the one that is most suitable for a specific scenario. Recognizing their complementary nature and synergistically integrating their strengths (for example, using DWT for initial denoising and then combining it with HHT for deep feature extraction) is the key to advancing PD signal analysis technology.

### 4.6. Signal Processing Based on Artificial Intelligence

In recent years, artificial intelligence (particularly deep learning) has become a research hotspot in partial discharge (PD) signal processing. Compared to traditional methods that rely on manually designed features (such as statistical features and wavelet packet decomposition), deep learning models such as Convolutional Neural Networks (CNNs), Recurrent Neural Networks (RNNs), and Long Short-Term Memory (LSTM) can automatically extract hierarchical, nonlinear features from large-scale data, significantly reducing the reliance on prior knowledge and complex feature engineering. This end-to-end learning paradigm demonstrates enhanced robustness in complex noisy environments, and multiple studies have demonstrated its ability to effectively improve classification accuracy and detection sensitivity across different PD types and signal-to-noise ratios [83,84,85]. The application of AI in PD signal processing primarily focuses on three core areas: efficient management of massive amounts of data, accurate identification of discharge patterns, and real-time diagnosis of system status.

#### 4.6.1. Compression and Data Management

Faced with the massive amount of data generated by partial discharge monitoring, AI technology has shown great value in data compression and management, providing innovative solutions to ensure the complete transmission, efficient storage and effective utilization of data.

AI-based lossy compression methods, such as autoencoders using skip connections and corrected data fusion, can achieve a compression ratio of up to 25:1 (compressing data to 4.1% of its original size) while still retaining key signal features for anomaly analysis [86]. Similarly, methods such as transmission sparse representation and double residual ratio thresholds achieve high-fidelity compression and reconstruction of PD signals by sparsely representing and accurately reconstructing noise components [87]. Studies have shown that this type of AI-driven new compression technology has significantly surpassed traditional methods in compression rate and effectively reduced the computational and storage complexity of back-end analysis systems [88].

At the data transmission and system management level, the combination of intelligent transmission algorithms with distributed server clusters, data concentrators, etc. can efficiently schedule and manage massive PD data, improve transmission efficiency while reducing system energy consumption, and demonstrate good engineering adaptability [89]. In addition, to address the common problems of sample imbalance and scarcity of labeled data in PD data, data augmentation technologies such as deep autoencoders based on generative adversarial networks can synthesize high-quality simulated samples, thereby enhancing the model’s ability to recognize the discharge patterns of minority classes and improving classification accuracy [90].

#### 4.6.2. Classification and Detection

The core advantage of AI in PD analysis lies in its powerful ability to classify and identify discharge patterns. Through machine learning and deep learning algorithms, AI can effectively distinguish different types of defects such as surface discharge and corona discharge, thereby accelerating and accurately locating the fault source. Taking convolutional neural networks (CNNs) as an example, they can automatically learn discriminative features using PD time-domain signals or phase-resolved partial discharge spectra (Phase-Resolved Partial Discharge (PRPD) spectra) as input. Literature reports that CNN can achieve a classification accuracy of 97.2% for various PD signals and a classification accuracy of over 93.8% for PRPD spectra for cable diagnosis, significantly outperforming traditional machine learning methods that rely on manual features [91,92]. In addition to high accuracy, AI models also exhibit excellent stability and can effectively reduce the false alarm rate of the detection system [93]. In addition, unsupervised learning techniques such as cluster analysis and hybrid models can be used to distinguish multi-source discharge defects and provide tools for reliability assessment of fault identification results [94].

#### 4.6.3. Real-Time Monitoring and Diagnostics

The integration of AI and real-time monitoring systems is driving the evolution of PD analysis from offline diagnosis to online intelligent early warning, greatly improving the fault response speed and operational reliability of power systems. Currently, AI algorithms have been successfully integrated into mobile monitoring platforms such as on-board distribution board diagnostic systems. Pattern recognition methods based on technologies such as fuzzy C-means (FCM) and radial basis function neural networks (RBFNN) have been verified in virtual simulation and actual operation environments, demonstrating their potential for application in rapid on-site diagnosis [95]. In addition, AI-driven solutions provide a new paradigm for the detection and diagnosis of cable faults, significantly improving the operation and maintenance efficiency of power systems [96].

Although AI has made significant progress in PD signal processing, it still faces many challenges in practical applications. First, the recognition accuracy of existing models still needs to be improved when distinguishing discharge types with similar physical mechanisms (such as surface discharge and corona discharge), which highlights the need to further optimize the model structure and feature learning capabilities [93]. Second, the performance of AI algorithms is highly dependent on data quality. Therefore, in actual deployment, reliable data acquisition and preprocessing processes are key prerequisites for ensuring the accuracy of analysis results [97]. In addition, the computational efficiency of the model, lightweight design to adapt to edge devices, and the interpretability of the decision process are all issues that must be addressed to achieve large-scale implementation of AI in industrial sites.

#### 4.6.4. Performance Comparison and Limitations

The reliable evaluation of AI model performance heavily relies on the datasets used. Current AI research in the field of power ionization (PD) faces a core challenge: the lack of large-scale, standardized, and widely accepted public benchmark datasets. This makes performance comparisons between different studies difficult and raises questions about the reproducibility and generalization ability of the results. Current research mainly relies on the following types of data:

Laboratory simulation data: PD signals generated in controlled environments using various defect models (such as pin plates and air gaps). This type of data has clear categories and a high signal-to-noise ratio, but may differ from real signals in complex field environments.

Proprietary field data: Field monitoring data from specific partner companies or projects, typically involving specific equipment such as transformers, GIS, and cables. While this type of data has real value, it is rarely publicly available due to commercial confidentiality or privacy reasons, and its scale is limited and annotation costs are high.

Limited public datasets: Currently available public datasets are relatively small in scale. For example, some studies may use datasets that only contain a few major discharge types (such as corona discharge, surface discharge, and internal discharge), with sample sizes ranging from several thousand to tens of thousands, which is considered small-scale datasets in the field of deep learning. And its scale and diversity still cannot compare with standard large datasets in fields such as computer vision. Table 7 shows the performance of some representative artificial intelligence algorithms in partial discharge signal analysis in recent years, and also indicates the source of the corresponding datasets.

Despite the immense potential of artificial intelligence in partial discharge diagnosis, its large-scale industrial application still faces several key challenges, with reproducibility and generalization ability being the core bottlenecks. First, at the data level, dataset variations lead to severe reproducibility and comparison difficulties. Due to the lack of standardized public benchmarks, most studies are trained and tested on private or self-built datasets. Differences in equipment type, defect morphology, signal-to-noise ratio, and sampling rate mean that a model performing well on one dataset may experience a sharp performance drop on another, making independent reproduction of literature results extremely difficult. Second, at the model level, insufficient generalization ability is a major obstacle to practical application. Diagnostic systems are typically trained under specific equipment or operating conditions, and their performance significantly degrades when faced with different equipment models, varying operating environments, or unknown discharge types. Models are more likely to learn shallow statistical regularities related to specific experimental settings rather than the underlying physical mechanisms of the discharge, limiting their universality. Furthermore, the inherent “black box” decision-making characteristics of deep learning result in a lack of physical interpretability. Models struggle to provide explanations related to the physical processes of the discharge that are convincing to domain experts. This lack of transparency constitutes a significant trust barrier when using diagnostic results for critical maintenance decisions. To address this fundamental issue, future research trends lean towards developing interpretable feature engineering based on physical mechanisms, embedding domain knowledge into model design to enhance the transparency and reliability of the decision-making process. Finally, the practical conflict between computational resources and embedded deployment remains prominent. A significant conflict exists between the high computational demands of complex models and the limited hardware resources and real-time processing requirements of field monitoring equipment. Lightweighting algorithms and efficient embedding on resource-constrained platforms remain pressing engineering challenges that need to be addressed.

#### 4.6.5. Emerging Frontiers: Edge AI and Embedded Detection

To address these challenges, particularly those related to computing resources and real-time performance, edge AI (Edge-AI) or embedded PD detection is emerging as a cutting-edge technology. Its core concept is to deploy lightweight AI models directly at the source of data collection, enabling local data processing and intelligent judgment. This not only significantly reduces reliance on communication bandwidth and cloud computing resources, aligning with the trend toward energy-efficient computing, but also significantly improves system response speed and privacy security.

Edge AI leverages the computing power of edge devices to process data locally, enabling real-time monitoring of power distribution units (PDs). This is crucial in remote substations or in inaccessible areas with limited internet connectivity, making traditional cloud solutions that rely on stable networks impractical. Specifically, by running deep neural networks locally on high-performance edge computing platforms (such as NVIDIA Jetson and Google Edge TPU), the system can minimize detection latency and power consumption, meeting stringent on-site energy efficiency requirements [100].

At the same time, embedded detection systems are deeply integrating AI models into dedicated hardware to achieve deeper, real-time monitoring and decision making. This is primarily achieved through two approaches:

- Hardware-Software Co-Design: System-on-Chip (SoC)-based solutions tightly integrate optimized AI models with hardware, enabling automatic generation of partial discharge (PD) alerts and long-term monitoring without human intervention [101].
- Microcontroller Deployment: Using dedicated tool chains such as STM32Cube. AI, models such as convolutional neural networks (CNNs) can be extremely lightweight and deployed on resource-constrained microcontrollers. This enables highly accurate, real-time PD identification on end devices, even under varying operating conditions [102].

Although edge artificial intelligence and embedded detection systems have brought revolutionary prospects to partial discharge monitoring, their widespread application in engineering practice still faces several key challenges. First, the efficient integration of artificial intelligence models into resource-constrained hardware platforms requires a delicate balance between computational complexity, power consumption, and real-time performance, which places extremely high demands on model lightweighting and hardware co-design. Second, the actual power operating environment is complex and changeable, and the systems deployed on-site must have strong robustness against noise interference and adaptability to different operating conditions, which poses a continuous test on the generalization ability and stability of the algorithm. Despite the above challenges, with the continuous evolution of edge computing architecture and innovative breakthroughs in lightweight artificial intelligence models, partial discharge detection systems based on edge intelligence are gradually showing stronger practicality and adaptability, and are expected to play an increasingly important role in the status perception and intelligent operation and maintenance of power equipment in the future.

### 4.7. Hybrid Partial Discharge (PD) Signal Processing Technology

Hybrid partial discharge (PD) signal processing technology aims to significantly improve the detection, denoising, and analysis capabilities of PD signals in electrical equipment by synergistically integrating digital and analog processing, advanced decomposition algorithms, and machine learning methods. This is crucial for accurate insulation diagnosis and fault warning. At the signal generation and front-end detection level, reconfigurable hybrid digital-analog systems can generate precise PD pulses with rise times less than 1 ns, whose spectra closely resemble the original PD signals. This provides a high-quality data foundation for subsequent machine learning diagnosis and reduces calibration complexity [103]. Similar fast hybrid systems, by combining analog and digital processing, achieve high sensitivity and resolution while effectively storing key data such as apparent charge and pulse timing for analysis and filtering out extraneous noise [104]. In the core denoising link, hybrid strategies have demonstrated strong advantages. For example, a method combining wavelet decomposition and singular value decomposition (SVD) can significantly improve the clarity of ultra-high frequency (UHF) PD signals, achieving a noise suppression ratio superior to traditional technologies, suitable for high-fidelity diagnosis of smart grid equipment [105]. Combining the complete integrated empirical mode decomposition with approximate entropy can effectively separate and reconstruct PD pulses submerged by strong noise [77]. In addition, a hybrid method for online monitoring of power transformers uses pre-whitening and blind equalization for noise suppression, and then achieves accurate PD pattern characterization through spectral conversion, which has been verified to be effective in both laboratory and field environments [106]. Hybrid approaches have been further developed at the advanced level of analysis and pattern recognition. An economical hybrid conversion scheme, leveraging peak detect-and-hold techniques and analog-to-digital conversion, provides a viable solution for online PD monitoring in small generators [107]. More cutting-edge, combining variational mode decomposition (VMD) with Choi–Williams distribution (CWD) time-frequency analysis, then inputting this into a hybrid convolutional neural network, achieves superior feature extraction and pattern recognition accuracy, effectively diagnosing insulation faults [108]. In signal classification and pulse recognition, a neural network-based approach that classifies pulse waveforms and compares them with reference patterns enhances the ability to distinguish different PD types [109]. A hybrid feature extraction method using adaptive optimal radial Gaussian kernel (AORGK) and two-dimensional non-negative matrix factorization (2DNMF) was used to classify partial discharge signals. This method achieved high classification accuracy, with a success rate exceeding 80% for fuzzy k-nearest neighbor (FKNN) and back-propagation neural network (BPNN) classifiers [110]. Looking at the hybrid techniques mentioned above, their core design logic lies in achieving complementary advantages through multi-level, multi-modal signal processing strategies to overcome the limitations of single methods. This integration typically follows this approach: first, a method (such as WT, EMD, or VMD) is used for signal decomposition or preliminary denoising to improve the signal-to-noise ratio; then, another method (such as SVD, approximate entropy, or time-frequency analysis) is employed to extract more discriminative features from the purified signal; finally, these features are input into a classifier (such as CNN or FKNN) or used for diagnostics based on their physical meaning. For example, the combination of WT and SVD leverages WT’s advantages in multi-scale analysis and SVD’s strengths in separating periodic noise; while the EMD/VMD + AI strategy utilizes the adaptive decomposition algorithm’s ability to handle nonlinear signals, providing better input features for AI models. Essentially, hybrid methods aim to achieve the optimal balance between noise suppression, feature enhancement, and classification accuracy throughout the entire chain of signal preprocessing, feature extraction, and pattern recognition. Despite the significant performance advantages of hybrid PD signal processing technology, its practical deployment, especially on resource-constrained edge devices, faces serious challenges in terms of real-time performance, power consumption, and hardware compatibility. Table 8 compares and analyzes typical hybrid strategies from an embedded deployment perspective.

In summary, the deployment of hybrid methods requires careful trade-offs between performance gains and engineering implementation costs. For stringent real-time online monitoring (latency < 100 ms), “WT + lightweight AI” is currently the most promising embedded hybrid solution. Hybrid methods involving adaptive decomposition algorithms such as EMD and VMD, due to their inherent computational complexity, are more suitable for latency-insensitive offline diagnostics or station-level edge server analysis under current technological conditions. In the future, designing hardware-friendly hybrid algorithms (such as fixed-point computation and approximation computation) and co-designing them with dedicated hardware (such as FPGAs and AI accelerators) will be key to driving high-performance hybrid technologies towards field applications.

## 5. Comparison

The preceding text systematically described various signal processing techniques used for partial discharge analysis, each with its unique theoretical foundation and methodological advantages. A critical synthesis of these methods reveals a clear path for technological development. The comparative analysis below first categorizes the methods based on their main advantages, providing a quantitative benchmark, followed by a qualitative synthesis to extract the core characteristics of each method family.

As summarized in Table 9, classifying PD signal processing methods according to their primary engineering objectives effectively reveals their performance landscape. Methods adept at noise suppression and signal enhancement (such as wavelet packet transform (WPT) and CEEMDAN) offer significant signal-to-noise ratio improvements (15–30 dB) and are computationally efficient, making them cornerstones of online monitoring [51,59,77]. In contrast, methods focused on high-precision classification and recognition (such as CNN and HHT) provide superior diagnostic accuracy (typically >92%) by utilizing learned or adaptive features [89,98]. Hybrid high-performance methods strategically integrate the first two categories, purifying the signal before advanced diagnostics, achieving the highest reporting accuracy (>98%) at the cost of increased computational complexity [108]. This performance-based classification provides concrete evidence for understanding the inherent trade-offs between accuracy, denoising capability, and computational cost.

Building on this quantitative overview, Table 10 provides a critical qualitative synthesis, further elucidating the fundamental principles, advantages, and limitations of each method family.

The comprehensive comparison in Table 10 provides a macro-level decision-making framework for selecting different signal processing technologies. It is worth noting that the performance evaluation of AI methods, particularly deep learning models, focuses more on different dimensions, such as data-drivenness, model architecture, and computing ecosystem. This has become a hot topic. Therefore, Table 11 summarizes the core application characteristics of AI methods over the past decade, hoping to provide a more targeted reference for technology selection and implementation.

According to Table 11, it is not difficult to see that, since 2022, the application of AI in the field of partial discharge has generally focused on using specific algorithms to optimize the process. This process can be the entire process of partial discharge from detection and signal acquisition to final results, or it can be aimed at optimizing a certain part of the process, such as the signal processing stage. Of course, due to the characteristics of AI, different algorithms have their own characteristics in performance optimization, anti-interference ability, and application scenarios. Comparing them can help clarify the focus of future research—that is, combining the advantages of multiple models to achieve a more comprehensive and robust PD monitoring system. Overall, these achievements have both differences and strong complementarity, providing rich reference and development foundation for the systematic application of AI in the field of partial discharge in the future.

### Unified PD Signal Processing Technology Selection Framework

Based on Section 5 quantitative and qualitative comparisons and the trends in AI, and to address the need for reproducible selection guidelines, we ultimately propose a unified evaluation framework. This framework recommends that practitioners prioritize their needs across three core dimensions based on their specific application scenarios: Diagnostic Accuracy, Computational Complexity, and Implementation Robustness.

1. Diagnostic Accuracy Demand: Assess the required level of fault discrimination.

Low priority/basic requirements: Focus on simple PD presence/absence detection, or rough differentiation of discharge types.

High priority/core requirements: Require fine identification and classification of complex PD patterns (e.g., distinguishing mixed discharge types), and pursue extremely high classification accuracy (>95%).

2. Computational Complexity Constraint: Evaluate the available computing resources and real-time requirements.

Strict Constraints/High Priority: Suitable for microcontroller deployment scenarios with strict limitations on power consumption and latency (e.g., response time <100 milliseconds required).

Relaxed Constraints/Low Priority: Suitable for cloud platforms or server processing, with no strict real-time requirements, allowing the execution of complex algorithm models.

3. Implementation Robustness Requirement: Assess the severity and complexity of the signal environment.

High Requirements/High Priority: Designed for environments with strong non-stationary noise, extremely low signal-to-noise ratios, or highly complex signal dynamics.

Low Requirements/Low Priority: Designed for controlled laboratory environments, high signal-to-noise ratios, and ideal conditions with relatively stable signals.

Based on the needs identified in the above dimensions, the following guidelines are provided for method selection:

Prioritize time-domain analysis and wavelet transform (WT): When computational complexity is strictly constrained (high priority), especially in online monitoring systems with extremely high real-time requirements.

Prioritize AI/deep learning methods: When diagnostic accuracy is the core objective of the project (high priority) and computational resource constraints are relatively relaxed.

Prioritize hybrid methods: When both robustness and diagnostic accuracy are high priorities, and sufficient computational resources are available.

Consider EMD/HHT methods: When dealing with highly nonlinear and non-stationary signals (a specific manifestation of high robustness requirements) and demanding high accuracy, but simultaneously seeking to avoid the black-box nature of AI models.

This framework transforms the method selection process from subjective experience-based judgment into a reproducible decision-making process based on multidimensional requirements analysis, thereby providing engineers with systematic guidance for selecting the most suitable PD signal processing method under specific constraints.

## 6. Gap Analysis and Future Prospects

**Figure 7:**
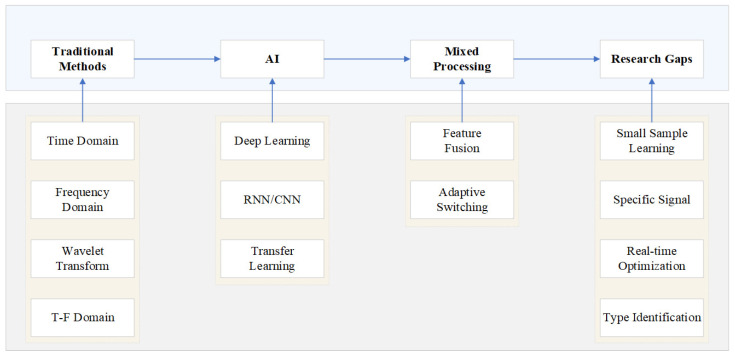
Evolution Path and Key Research Gaps in Partial Discharge Detection Technology.

Currently, AI-based partial discharge (PD) signal processing research is evolving from general pattern recognition to precise, deployable diagnostic systems. However, critical technical gaps remain in achieving the ultimate goal of high-precision classification based on specific physical characteristics of discharges and effectively deploying them in resource-constrained embedded devices. These challenges can be clearly summarized as the research gaps shown in Figure 7.

### 6.1. Key Research Gaps

Insufficient fine-grained feature extraction and decoupling capabilities for classification. When processing mixed PD signals, existing AI models (such as CNNs) struggle to reliably decouple and extract truly effective microscopic features strongly associated with specific discharge types (such as specific oscillation patterns associated with internal discharges or precise frequency centers associated with surface discharges) from complex background noise and the superposition of multiple discharge sources. These models are more likely to learn superficial statistical patterns in the data rather than the underlying physical characteristics, resulting in a high rate of misclassification of similar discharge types (such as surface discharge and corona discharge). For example, existing models may fail to reliably decouple and extract microscopic features strongly correlated with specific discharge types, such as the persistent oscillation patterns of surface discharge in the 300–800 MHz frequency band, or the pulse clusters with specific phase distributions that appear near the voltage peak in corona discharge [14]. Models are more likely to learn surface statistical regularities in the data than underlying physical characteristics, leading to a persistently high misclassification rate for similar discharge types [111].

The contradiction between high-precision real-time classification and resource constraints on embedded devices. While some research has attempted to deploy AI models on edge devices, these models are often overly simplified to meet the computing power, memory, and power consumption constraints of embedded platforms (such as the STM32 and Jetson Nano), resulting in significantly lower classification accuracy and robustness than cloud-based models. Currently, there is a lack of end-to-end, lightweight solutions that can perform feature extraction, classification, and localization of multi-source PD signals on embedded systems within microseconds.

The lack of standardized PD classification datasets and benchmarks for embedded platforms remains a critical obstacle. To effectively train and evaluate embedded AI models, such datasets must go beyond simply containing pure PD signals and comprehensively simulate real-world embedded system scenarios. This includes incorporating specific sampling rates (e.g., 100 MS/s), quantization precision (e.g., 12 bits), channel counts, and crucially, typical noise characteristics of embedded systems (e.g., power supply ripple, quantization noise). Furthermore, a truly valuable benchmark should cover various discharge types generated by different devices under diverse operating conditions and signal-to-noise ratios. Currently, the severe shortage of such standardized resources hinders fair comparison and effective iteration of algorithms, constituting a key bottleneck in establishing a reliable closed-loop system from “signal source” to “embedded device.”

### 6.2. Discussion and Recommendation

To fill the research gap and promote PD classification technology from the laboratory to industrial embedded applications, future research should focus on the following directions:

- Develop embedded, interpretable feature engineering based on physics mechanisms: Abandoning the single-minded “end-to-end black box” approach, we instead design a lightweight feature extraction front-end that incorporates prior physics knowledge of PD. For example, feature vectors of atomic or physical information in the time-frequency domain strongly associated with specific discharge types are computed in real time on the embedded side and then fed into a small classifier. This not only improves the model’s interpretability for classifying specific PD sources but also significantly reduces computing resource requirements, directly addressing the challenge of “intelligent feature extraction and decoupling.”
- Build specialized lightweight network architectures for embedded classification: Explore asymmetric neural network architectures, spiking neural networks, or attention-based dynamic inference networks optimized for PD signal classification. These models should dynamically allocate computing resources based on the complexity of the input signal, prioritizing the ability to distinguish key PD types. This approach achieves an optimal balance between accuracy and efficiency within the strict constraints of embedded platforms, resolving the core challenge of high-precision, real-time classification on embedded devices.
- Establish an open benchmark and simulation-measurement closed loop for embedded PD classification: Create an open-source, large-scale embedded PD classification benchmark dataset (e.g., Emb-PD-1.0) containing multi-source PD signals from different devices, sampling settings, and noise levels. Simultaneously, develop embedded hardware-in-the-loop simulation technology to allow algorithms to be fully tested and validated in a virtual embedded environment before deployment. This will form a rapid, iterative “design-simulation-deployment” closed loop, accelerating the maturity of high-performance embedded classification solutions. This initiative will directly address the lack of standardized datasets and benchmarks.

By deepening our research in these areas, we hope to eventually bridge the technological gap shown in Figure 7 and achieve accurate, rapid, and low-power automatic identification and classification of specific PD signal sources on the device side, providing truly online intelligent perception capabilities for predictive maintenance of power equipment.

## 7. Conclusions

This article systematically reviews advanced methods for partial discharge (PD) signal processing, charting the evolution from traditional time-frequency analysis to modern artificial intelligence (AI) and hybrid technologies. This review demonstrates a clear trend in this field, transitioning from general-purpose signal processing to accurate, interpretable, and deployable diagnostic solutions.

Key insights can be summarized as follows: Traditional methods (such as time-domain analysis, Fourier transform, wavelet transform, and HHT) lay the foundation for PD analysis, and their computational efficiency and physical intuitiveness remain valuable in specific scenarios. However, they have inherent limitations in handling complex noise and enabling automated diagnosis. Artificial intelligence, particularly deep learning models, has achieved breakthroughs in classification accuracy through its powerful end-to-end feature learning capabilities. However, their engineering applications remain constrained by their reliance on large-scale annotated data, the “black box” nature of model decision making, and high computational overhead.

Looking forward, this review highlights a crucial frontier: the development of physics-informed learning frameworks for embedded intelligent diagnosis. Future research must move beyond general pattern recognition and focus on achieving accurate and interpretable classification based on the specific physical mechanisms of the discharge source on resource-constrained embedded platforms. This requires a new generation of algorithms that deeply integrate discharge physics priors with lightweight network design, enabling direct extraction and reliable identification of the essential characteristics of specific PD signals at the edge.

In summary, the next leap forward in partial discharge signal processing technology will depend on whether we can successfully build intelligent diagnostic systems that not only deeply understand the physical nature of specific PD signals but also operate efficiently at the edge. Cross-disciplinary innovation, particularly the deep integration of signal processing, solid-state electrical physics, and edge computing technologies, will be key to achieving this goal.

## Figures and Tables

**Table 1:** Lists the signal characteristics and processing methods of these discharge types.

Partial Discharge Type	Signal Characteristics	Corresponding Treatment Methods
Corona Partial Discharge	With different time-frequency characteristics	It can be used for effective filtering and identification [14,15].
Internal Partial Discharge	It has a stronger low-frequency component, a longer duration and a wider spectrum.	Bandpass filtering reduces interference, and then wavelet transform is used to extract time-frequency features, and finally recognition is performed [16].
Surface Partial Discharge	Its frequency center is approximately between 30 MHz and 800 MHz.	Acoustic emission technology and wavelet transform are usually used for analysis to perform effective classification [15,17].
Gap discharge	Usually high frequency and random	Advanced signal processing techniques such as wavelet analysis and machine learning are required for accurate recognition [18,19].

**Table 2:** Commonly used time domain characteristic parameters of partial discharge signals.

Parameter Category	Characteristic Parameters	Advantage	Disadvantage
Basic amplitude characteristics	Peak—The maximum amplitude of a pulse (voltage or current) in the time domain. Average Amplitude—The arithmetic average of the amplitudes of all sampling points within a pulse or a signal.	- Simple calculations - Intuitive physical meaning	- Extremely poor noise immunity - Low discrimination
Pulse morphology characteristics	Rise Time—The time required for a pulse to rise from 10% to 90% of its peak value. Fall Time—The time required for a pulse to fall from 90% to 10% of its peak value. Pulse Width—The full width of the pulse at 50% of its peak value. Overshoot and Ringing—The amplitude of the reverse peak following the main peak of the pulse or the presence of decaying ringing.	- High discrimination - Good robustness	- High computational complexity - Very sensitive to measurement system bandwidth
Statistical distribution characteristics	Skewness describes the asymmetry of the pulse amplitude distribution. A positive skewness indicates a longer tail on the right (high amplitude) side. Kurtosis describes the steepness of the distribution of pulse amplitudes. A higher kurtosis means the distribution is more concentrated and has more prominent tails.	- Strong macroscopic characterization capabilities. - A certain degree of suppression of random noise.	- Loss of timing information - Low specificity

**Table 3:** Processing methods and applications corresponding to time domain characteristic parameters.

Method	Parameter	Application	Reference
Amplitude threshold method	- Peak - Value/Amplitude	The adaptive amplitude thresholding method based on wavelet coefficients can automatically determine the optimal threshold using the background noise signal before PD occurs as a reference. Hard thresholding effectively suppresses noise interference while preserving the amplitude characteristics of PD pulses.	[22,23]
Pulse waveform identification method	- Rise time - Fall time - Pulse width - Oscillation	Based on the feature extraction of time-domain pulse waveform and its probability distribution, clustering and classification diagnosis of local discharge sources can be achieved.	[24,25]
Statistical feature extraction	- Skewness - Kurtosis - Shape factor - Pulse factor	In power cable systems, statistical parameters such as mean, skewness, and kurtosis are used to plot PD fingerprints, which help in identifying different types of defects.	[26,27]

**Table 4:** Performance comparison of Fourier Transform and its variants in PD analysis.

Category	Principle	Advantage	Disadvantage	Application/Reference
FFT	Globally transform the entire signal from the time domain to the frequency domain.	- Accurate frequency domain positioning, enabling clear extraction of stable frequency components. - Mature algorithm, widely used.	- Completely loses time domain information, making it impossible to analyze frequency occurrence. - Applicable only to stationary signals, with limited ability to analyze PD transient pulses.	Noise cancelation and signal filtering [33]. Suppression of narrowband and discrete spectral interference [34,35]. Time-frequency analysis and signal characterization [45]. Integration with advanced technologies [40]. Limitations and complementary approaches [40,45].
STFT	By sliding a fixed time window, FFT is performed on the signal segments to provide time-frequency analysis.	- Provides limited time-domain and frequency-domain information simultaneously. - Visually displays how frequency components change over time. - Suitable for analyzing transient characteristics.	- Fixed time-frequency resolution, constrained by the Heisenberg uncertainty principle. - Difficulty in simultaneously capturing rapidly changing transient pulses and sustained oscillations.	Time-frequency analysis [36,46]. Signal processing and noise reduction [36,42]. Comparative analysis with other technologies [43,45]. Online monitoring system for high voltage cables [47].
LPFT	An extension of the STFT that improves time-frequency aggregation through polynomial modeling.	- Compared to STFT, it has higher time-frequency resolution and accuracy. - It has a significant advantage in revealing details in low- and mid-frequency components.	- High computational complexity. - Sensitive to the degree of signal model matching.	Enhanced frequency component detection [+–45]. Application in distribution transformers [39]. Applications in various signal processing scenarios [44].
FRFT	A generalized form of the Fourier transform that transforms a signal into the fractional domain between time and frequency.	- Provides a new dimension for analyzing non-stationary signals. - Provides enhanced analytical capabilities for certain types of signals (such as linear frequency modulation).	- The physical concepts are abstract and difficult to understand and apply. - Choosing the optimal fractional order is challenging. - The scope of application is relatively specific and not a universal tool.	The spectral decomposition of the partial discharge measurement signal is performed by jointly applying short-time Fourier transform (STFT) and singular value decomposition (SVD) [48].

**Table 5:** Comparison of EMD, Ensemble Empirical Mode Decomposition (EEMD) and Complete Ensemble EMD with Adaptive Noise (CEEMDAN) methods.

Feature Dimension	EMD	EEMD	CEEMDAN
Principle	An adaptive signal decomposition method that decomposes a complex signal into a series of intrinsic mode functions (IMFs) through a “sieving” process.	By adding Gaussian white noise to the original signal multiple times and performing EMD, the influence of noise is eliminated by ensemble averaging to suppress modal aliasing.	Based on EEMD, specific white noise is adaptively added during each order of IMF decomposition, and the residual is calculated by ensemble averaging, which can better reconstruct the signal and reduce the noise residue.
Advantage	Fully adaptive, no basis functions required. Highest computational efficiency. Suitable for nonstationary and nonlinear signal analysis.	Significantly reduces modal aliasing. More stable than EMD, resulting in more unique results. Preserves the adaptability of EMD.	Almost completely eliminates modal aliasing. Extremely low signal reconstruction error and excellent integrity. Fewer integration steps are required, resulting in higher computational efficiency than EEMD.
Disadvantage	The modal aliasing problem is serious and it is sensitive to noise.	The computation is large and there is residual noise.	The computational effort is still greater than that of the original EMD and the algorithm implementation is more complex.
Scope of application	Preliminary analysis of PD signals with high signal-to-noise ratio and relatively simple signal components. And preliminary exploration of online monitoring systems with high real-time computing requirements.	Processing PD signals containing complex mixed noise (such as white noise and narrowband interference). Scenarios requiring stable and reliable feature extraction for pattern recognition.	High-precision analysis and complete signal reconstruction are required. It is also suitable for processing weak PD signals or signals with very complex components and similar time scales.

**Table 6:** Comprehensive comparison of time-frequency analysis methods (WT, EMD, HHT).

Comparison Dimension	Wavelet Transform	EMD	Hilbert–Huang Transform
Core Principles and Basis Functions	Linear projection based on a predefined fixed wavelet basis.	No basis function, adaptive “sieving” decomposition based on the data itself.	EMD + Hilbert transform, adaptive time-frequency representation based on IMF.
Decomposition structure and resolution	Regular “pyramid” fixed structure; fixed resolution, constrained by the uncertainty principle.	Irregular, adaptive IMF sequence; adaptive resolution, high temporal resolution at signal drastic changes.	Based on the irregular structure of EMD; output time-frequency spectrum with adaptive high resolution.
Robustness and computational efficiency	High. The algorithm is mature and stable, with high computational efficiency and strong noise and modal aliasing resistance.	Moderate. Sensitive to noise and intermittent signals, prone to modal aliasing; moderate computational effort, lacks rigorous theory.	Low. The computational complexity is high, the robustness is limited by the EMD step, and the physical meaning of the instantaneous frequency is controversial.
Role and value in PD analysis	Powerful pre-processor/filter: Suitable for online monitoring, real-time noise reduction, and PD pulse extraction and positioning, with advantages in efficiency and stability.	Adaptive signal decomposer: It excels at processing nonlinear and non-stationary signals and is often used as the front end of hybrid methods for exploratory analysis.	Fine-grained feature extractor: Provides high-resolution time-frequency energy distribution, excellent fault classification and diagnosis capabilities, and is suitable for offline in-depth analysis.

**Table 7:** Performance comparison of artificial intelligence models in PD analysis.

Model Architecture	Main Input	Reporting Accuracy/Performance	Types-Dataset Used and Key Features	Applicable Scenarios
Convolutional Neural Networks (CNNs)	PRPD spectrum, time domain signal waveform.	Accuracy rates as high as 93.8–97.2% [91,93].	Proprietary Data from Amar Telidji university of Laghouat and HD Hyundai Electric Co., Ltd. and KETEP KOREA-The data combines laboratory and field data, including various defect models such as corona discharge, surface discharge, and internal discharge.	High-precision classification and recognition, especially suitable for processing PRPD spectra with image structure.
Recurrent Neural Networks (RNNs/LSTM)	Time domain signal sequence.	Classification accuracy of phase-resolved partial discharge (PRPD) signals in gas-insulated switchgear (GIS) is 96.74% [98].	Proprietary Data from lab and Korea Electric Power Corporation-GIS equipment-specific field data focuses on capturing timing characteristics related to the phase of power frequency voltage.	Analyze the time evolution of PD pulses and sequence-dependent fault diagnosis.
Generative Adversarial Networks (GANs)	A small amount of real PD data.	Successfully generated high-quality samples to improve classification [90].	Proprietary Data-Small sample laboratory datasets are used to address the problems of data scarcity and class imbalance.	Data augmentation improves model robustness in small-sample learning and imbalanced datasets.
Autoencoder	Original PD signal.	Compression ratio up to 25:1 [86,99].	Public Data-Time series data of noise from overhead transmission lines, including anomalous signals in the real environment.	Data compression and anomaly detection reduce the burden on back-end analysis systems.

**Table 8:** Feasibility analysis of embedded deployment of typical hybrid PD processing methods.

Hybrid Strategy	Expected Inference Delay	Hardware Limitations and Deployment Feasibility	Key Bottleneck
WT+ Lightweight AI Classifier	Medium to High (~10 ms–100 ms)	The feasibility is relatively high. WT can be implemented on low-end MCUs; lightweight AI (such as SVM, decision tree) or quantized miniature CNNs can be deployed on high-end MCUs (such as ARM Cortex-M7) or edge SoCs (such as Jetson Nano).	The complexity and memory footprint of AI models are the main limitations. Increasing the number of decomposition layers in WT. significantly increases the computational cost.
EMD/EEMD + AI Classifier	High (~100 ms–seconds)	Low feasibility. The iterative and interpolation processes of the EMD algorithm are computationally intensive, making it difficult to meet real-time requirements on microcontrollers. It typically needs to run on an embedded CPU (such as the Cortex-A series) or a higher-level processor.	The decomposition process of EMD intrinsic mode function (IMF) is the main source of delay, and its computational complexity increases nonlinearly with signal length and complexity.
VMD + AI Classifier	Medium to High (~50 ms–500 ms)	The feasibility is moderate. VMD is generally more computationally efficient than EMD, but it remains complex. It requires the support of a high-performance edge computing platform (such as Jetson TX2, Google Edge TPU).	Optimizing the number of VMD iterations and modalities is crucial for achieving real-time processing.
CEEMDAN + Feature Extraction	Very high (seconds and above)	The feasibility is extremely low. CEEMDAN suppresses modal mixing through multiple ensemble averaging, which incurs huge computational overhead and is only suitable for offline cloud analytics.	The number of times the averaging is integrated directly determines the computational cost, which cannot meet the latency requirements of real-time on-site monitoring.

**Table 9:** Typical performance of different PD signal processing methods in core performance dimensions.

Method Category	Core Strengths and Focus	Representative Technologies	Key Performance Indicators	Computational Complexity/Applicable Scenarios
Noise suppression and signal enhancement	Extracting and enhancing PD pulses from strong background noise	Wavelet thresholding for noise reduction	Signal-to-noise ratio improvement: 15–25 dB	Low cost/Suitable for online preprocessing and embedded systems
		Wavelet Packet Transform (WPT)	Signal-to-noise ratio improvement: 20–30 dB	Medium/Suitable for edge computing and fine noise reduction
		CEEMDAN and Approximate Entropy	It can effectively separate PD signals under strong noise background.	High/Suitable for offline analysis and extremely low signal-to-noise ratio scenarios
High-precision classification and recognition	End-to-end identification of discharge type and fault mode	CNN (PRPD spectral input)	Classification accuracy: 93–97%	Training: Very High Inference: Medium/Cloud or High-Performance Edge Server
		RNN/LSTM (Time-series signals)	Classification accuracy: 95–97%	Training: Very High Inference: Medium/Cloud or High-Performance Edge Server
		HHT (EMD + Hilbert spectrum)	Classification accuracy: 92–96%	High performance/Suitable for offline diagnosis and feature analysis
Comprehensive high-performance hybrid	By combining multiple techniques, a globally optimal solution from denoising to classification can be achieved.	WT/EMD + AI Classifier	Classification accuracy: 96–99% (Improved through pre-processing noise reduction)	Medium to high/dependency preprocessing chain, suitable for high reliability diagnostics
		VMD + Time-Frequency Analysis + CNN	Classification accuracy: >98%	Very high/Suitable for offline, high-precision diagnostic systems

**Table 10:** Comprehensive comparative analysis of PD signal processing technologies.

Method	Principles/References	Advantages	Limitations/Challenges	Ideal Application Scenarios
Time domain analysis	Direct analysis of pulse parameters (amplitude, rise time, statistical moments, etc.).	- Intuitive and simple calculations. - Effective for pulse identification and initial trend analysis.	- Poor noise immunity. - Loss of all frequency information. - Limited feature set for complex patterns.	Preliminary screening and real-time pulse counting under high signal-to-noise ratio conditions.
Fourier transform and variants (FFT, STFT, LPFT, FRFT)	Global (FFT) or windowed (STFT) projection onto sine basis functions.	- Excellent frequency localization capabilities (FFT). - Provides time-frequency insights (STFT).	- Fixed resolution (STFT, subject to Heisenberg’s uncertainty principle). - Predefined basis may not match PD transient signals. - Insufficient ability to handle nonlinear signals.	Identify stable resonant frequencies (FFT) and analyze quasi-stationary transient signals (STFT/LPFT).
Wavelet Transform (WT)	Multiresolution analysis using a scalable and translatable wavelet basis.	- Adaptive time-frequency resolution (high temporal resolution at high frequencies). - Powerful denoising capabilities through thresholding. - Flexible basis function selection.	- Performance is highly dependent on parameter selection (mother wavelet, number of decomposition levels, threshold). - Computational complexity can be high.	The de facto standard for non-stationary PD pulse denoising and analysis. The preferred method for robust feature extraction.
Empirical Mode Decomposition (EMD) and variants (EEMD, CEEMDAN)	Data-driven, adaptive decomposition into intrinsic mode functions (IMFs).	- Fully adaptive, no predefined basis required. - Effective for nonlinear and nonstationary signals. - Its variant (CEEMDAN) exhibits excellent noise separation capabilities.	- Modal aliasing (raw EMD). - Computationally expensive (EEMD, CEEMDAN). - Sensitive to noise and stopping criteria.	It can process complex nonlinear signals for which wavelet basis is not applicable. When combined with other methods, it is effective in extremely low signal-to-noise ratio scenarios.
Hilbert–Huang transform (HHT)	EMD is followed by Hilbert transform to obtain the instantaneous frequency.	- Provides high-resolution time-frequency spectrum. - Excellent performance in feature extraction and pattern recognition.	- Inherits the drawbacks of EMD (modal mixing, sensitivity). - The definition of instantaneous frequency may not be physically clear.	When detailed time-frequency energy mapping is required for fine pattern analysis and fault diagnosis.
Artificial Intelligence/Deep Learning	End-to-end feature learning from raw data or preprocessed input (such as PRPD spectrograms).	- Automatic, hierarchical feature extraction. - Achieve state-of-the-art accuracy in classification and detection. - Robust to complex noise patterns.	- Requires a large labeled dataset. - High computational cost during the training phase.	Large-scale condition monitoring systems with massive historical data aim to achieve automated classification and high accuracy.
Hybrid methods	The above technologies are strategically integrated to overcome the limitations of a single approach.	- Synergistic effects, such as WT/EMD denoising + AI classification. - Achieve performance unattainable by any single method. - Enhanced robustness and accuracy.	- Highest design and implementation complexity. - More challenging to tune parameters.	At the forefront of research. Ideal for mission-critical applications, extreme noise environments, and wherever the highest diagnostic confidence is required.

**Table 11:** Application of AI in partial discharge from 2022 to date.

Reference	Insights	Core
[18]	The AI algorithm utilizes the complete PD current waveform combined with advanced compression technology to improve data compression rate, simplify analysis systems, and achieve efficient automatic partial discharge diagnosis in AC and DC high-voltage systems.	This study investigated the compression method of high-resolution partial discharge current waveforms based on artificial intelligence in AC and DC high-voltage systems, achieving significantly higher compression rates and simplifying AI based analysis systems.
[86]	This study proposes a lossy compression method for partial discharge in overhead power lines, which effectively compresses PD signals using an autoencoder with skip connections, with a compression ratio of approximately 25, improving remote monitoring and fault diagnosis capabilities. This has laid the foundation for future fault detection.	This article proposes a new lossy compression method that utilizes an autoencoder with skip connections and corrected data to achieve a compression factor of 25 times while retaining the basic characteristics of local discharge monitoring in transmission systems.
[88]	This article explores the use of AI based compression methods to achieve high-resolution PD current waveforms in AC and DC high voltage systems. It shows that utilizing the entire waveform has advantages over traditional indicators, improves compression rates, simplifies analysis systems, and enhances the efficiency of automatic partial discharge detection.	This study investigates the compression method of high-resolution partial discharge current waveforms based on artificial intelligence in AC and DC high-voltage systems, achieving significantly higher compression rates and simplifying AI based analysis systems.
[90]	The DAE-GAN proposed in this article, combined with an autoencoder, enhances PD pattern recognition by generating real samples in limited and imbalanced data, significantly improving recognition accuracy compared to other algorithms.	The proposed DAE-GAN enhances pattern recognition capability by improving probability distribution fitting and improving recognition accuracy under limited and imbalanced sample conditions, generating more realistic partial discharge samples.
[91]	This article proposes a CNN based PD signal classification method, which has achieved an accuracy of 97.2% on corona, surface, and internal PD datasets through preprocessing and architecture optimization, outperforming traditional methods. The excellent performance of indicator analysis and error classification research provides a basis for future improvement.	This study proposes an automatic partial discharge classification method based on CNN, with an accuracy of 97.2% and better performance than traditional methods. It has had an impact on state monitoring and practical applications in high-voltage engineering and power systems.
[93]	This study utilized CNN to analyze PRPD images, improving the accuracy of cable partial discharge diagnosis to over 93.8%, demonstrating the potential of AI in predicting maintenance and improving infrastructure reliability.	This study applies artificial intelligence to improve PRPD pattern recognition in power cables, achieving an accuracy of 93.8% through a CNN model. Predictive maintenance is achieved through defect classification and diagnosis, and the operational efficiency of power companies is improved.
[94]	This article analyzes a deep learning tool based on PRPD mode for detecting partial discharge, compares the performance of three high-precision models under complex noise and defect conditions, and evaluates their effectiveness in fault recognition and power grid monitoring using critical matrices.	This article explores the application of deep learning tools in automatic partial discharge detection based on PRPD mode. Three data models were implemented and compared using different architectures and training datasets. The characteristics of the model aim to evaluate its performance under practical conditions, including noise mixed with defects and clustering techniques used to separate multiple defects.
[95]	The main achievements of this study include extracting features from partial discharge signals, optimizing models to improve accuracy, and performing multimodal recognition across different domains. This study emphasizes the importance of these advances in improving the reliability and safety of power systems, while also pointing out the potential for future development in this field.	In this article, a large amount of experimental data and long-term on-site operational experience indicate that partial discharge (DC) is the main cause of insulation system damage and power outages in electrical equipment. Therefore, strengthening effective detection of local DC is a necessary measure to ensure the safe and reliable operation of power systems.
[96]	This article proposes a real-time partial discharge monitoring system for airborne switchboard based on FCM-RBFNN, using HFCT sensors to collect data, and combining PRPS and PRPD analysis to verify the excellent performance of the model in both virtual and real environments.	This article proposes a vehicle mounted switchboard diagnostic system based on the Ai algorithm, with the aim of establishing a real-time partial discharge monitoring and diagnostic system. However, Ai compared twice in total.
[97]	This article proposes the use of CNN to analyze partial discharge in transmission cables, based on the MCSG-PD-6016 dataset. The detection accuracy is 81–94%, and the recall rate is 83–96%, demonstrating its stability and potential in cable fault detection and promoting the application of deep learning in power systems.	The accuracy of the model proposed in this study remains stable at a high level, with excellent recall performance, fully demonstrating the effectiveness of deep learning in analyzing cable partial discharge signals.

## Abbreviations

The following abbreviations are used in this manuscript:

AI	Artificial Intelligence
AORGK	Adaptive Optimal Radial Gaussian Kernel
BPNN	Back-Propagation Neural Network
CEEMDAN	Complete ensemble EMD with adaptive noise
CNN	Convolutional Neural Network
CWD	Choi–Williams Distribution
DWT	Discrete Wavelet Transform
EEMD	Ensemble Empirical Mode Decomposition
EMD	Empirical Mode Decomposition
FFT	Fast Fourier Transform
FKNN	fuzzy k-nearest neighbor
FRFT	Fractional Fourier transform
FT	Fourier Transform
GAN	Generative Adversarial Network
HHT	Hilbert–Huang Transform
IMF	Intrinsic Mode Functions
LPFT	Local polynomial Fourier transform
PD	Partial Discharge
RNN	Recurrent Neural Network
STFT	Short-time Fourier transform
SVD	Singular Value Decomposition
VMD	Variational Mode Decomposition
WPT	Wavelet Packet Transform
WT	Wavelet Transform
